# Phenolic Acids of Plant Origin—A Review on Their Antioxidant Activity In Vitro (O/W Emulsion Systems) Along with Their in Vivo Health Biochemical Properties

**DOI:** 10.3390/foods9040534

**Published:** 2020-04-24

**Authors:** Sotirios Kiokias, Charalampos Proestos, Vassiliki Oreopoulou

**Affiliations:** 1 Research Executive Agency (REA), Place Charles Rogier 16, 1210 Bruxelles, Belgium; Sotirios.KIOKIAS@ec.europa.eu; 2 Laboratory of Food Chemistry, Department of Chemistry, National and Kapodistrian University of Athens, Panepistimiopolis Zografou, 15784 Athens, Greece; harpro@chem.uoa.gr; 3 Laboratory of Food Chemistry and Technology, School of Chemical Engineering, National Technical University of Athens, Iron Politechniou, 9, 15780 Athens, Greece

**Keywords:** phenolic acids, emulsions, antioxidants, health properties

## Abstract

Nature has generously offered a wide range of herbs (e.g., thyme, oregano, rosemary, sage, mint, basil) rich in many polyphenols and other phenolic compounds with strong antioxidant and biochemical properties. This paper focuses on several natural occurring phenolic acids (caffeic, carnosic, ferulic, gallic, p-coumaric, rosmarinic, vanillic) and first gives an overview of their most common natural plant sources. A summary of the recently reported antioxidant activities of the phenolic acids in o/w emulsions is also provided as an in vitro lipid-based model system. Exploring the interfacial activity of phenolic acids could help to further elucidate their potential health properties against oxidative stress conditions of biological membranes (such as lipoproteins). Finally, this review reports on the latest literature evidence concerning specific biochemical properties of the examined phenolic acids.

## 1. Introduction and Target of This Review

By “plant phenolics”, we refer to a wide range of natural compounds (e.g., anthocyanins, flavonoids, phenolic acids etc.) with varying structural characteristics that modulate their antioxidant activity and their subsequent health and biological effects [1,2].

The phenolic acids, in particular, offer an important group of powerful natural compounds that having substantial lipid and water solubility can inhibit oxidative deterioration when added as functional ingredients in emulsion model systems [3]. Research studies in this field have focused on the free radical scavenging capacity of phenolic acids and more specifically on radical quenching that can be validly measured through kinetic parameters [4]. Over the last decade, the food industry has increasingly considered the use of natural phenolic antioxidants as an efficient strategy to retard oxidative deterioration in food-based systems and thereby maintain their sensory characteristics [5,6].

In emulsified foods, (e.g., dressings, sauces, soups, and desserts) lipid oxidation can occur rapidly due to their large surface area [7,8] with mechanisms that are more complex and not fully understood compared to bulk oils [9,10]. Furthermore, the antioxidant activity in interfaces can be crucial not only for developing novel food applications but also for further exploring their potential health properties against oxidative stress [11]. Emulsion systems generally mimic the amphiphilic nature and the basic structural characteristics of important biological membranes (e.g., lipoproteins) that are prone to oxidative degradation when attacked by singlet oxygen and free radicals [12]. A typical example of a “bio-interfacial system” is offered by plasma lipoproteins, which are complex aggregates of lipids and proteins that render the hydrophobic lipids accessible by the aqueous environment of body fluids and thereby by reactive oxygen species potentially present [13]. The initiation of these harmful biochemical processes leads to in vivo oxidative damage of biomolecules and ultimately the development of serious human health conditions such as aging, carcinogenesis, and cardiovascular diseases [14,15].

This paper first examines in Section 2 the most common naturally occurring phenolic acids that can be extracted from various plant sources, including edible herbs and well-known botanicals. In addition, the authors provide a summary of the available research findings concerning the antioxidant activity of each examined phenolic acid in o/w emulsion systems. Overall, in vitro research on the oxidative stability and antioxidant effects of phenolic acids in model emulsions could provide useful background knowledge and information of nutritional interest to support in vivo clinical trials.

In the last few years, an increasing body of clinical research has focused on the potential effects of phenolic acids against the development of cancer, cardiovascular diseases, and other health disorders (such as skin problems, inflammations, bacterial infections etc.). The main target of this publication is to provide in Section 3 an overview of the most recent literature evidence concerning the health beneficial effects of the examined phenolic acids.

## 2. Natural Sources and Antioxidant Activities of Phenolic Acids in Food Emulsions

This section provides a summary of the most important natural sources for a few common—in nature and food—phenolic acids that are listed along with their chemical structure in Table 1. In parallel, the authors have performed a literature search on the in vitro antioxidant activities of each examined phenolic acid against the oxidation of oil-based emulsion systems.

### 2.1. Caffeic Cid (CA)

CA is a hydroxycinnamic acid structurally composed of both phenolic and acrylic functional groups, the derivatives of which are *trans* in nature [16,17]. Ιt is found at high levels in some herbs, especially in the South American herb yerba mate (1.5 g/kg) [18]*,* and thyme (1.7 mg/kg), [19]. In fruits (such as berries, apples, and pears) CA was quantified in high amounts, representing together with p-coumaric acid 75–100% of the total hydroxycinnamic acids [20]. Also, CA can be found in the bark of *Eucalyptus globulus* [21] and was identified as the main phenolic constituent in coffee and coffee oil [22]. Boke et al. (2019) [23] analysed samples of *Cephalaria* species using high-performance liquid chromatography coupled with tandem mass spectrometry (HPLC-MS/MS) and determined CA as major phenolic acid.

A few researchers have reported a clear antioxidant effect of CA in various Tween-based emulsions prepared with linoleic acid [4] or other vegetable oils such as corn, flaxseed, and sunflower oils [24]. Sorensen et al. (2017) [25] observed that CA presents a clear antioxidant activity in Citrem-and Tween-stabilised emulsions in the presence of endogenous tocopherols but acted as a prooxidant in the absence of tocopherols. The authors suggested that the observed differences in antioxidant efficiency with different emulsifiers (and with or without endogenous tocopherols) were caused due to emulsifier–antioxidant interactions and antioxidant–antioxidant interactions in the emulsions.

### 2.2. Gallic Acid (GA)

GA (also known as 3,4,5-trihydroxybenzoic acid) is the main phenolic acid in tea [26] but also found in high amounts in chestnuts and several berries [19]. It is encountered in a number of land plants, such as the parasitic plant *Cynomorium coccineum*, the aquatic plant *Myriophyllum spicatum*, and the blue-green alga *Microcystis aeruginosa* [27,28]. Very recently, Souza et al. (2020) [29] has isolated gallic acid from black tea extract at a concentration around 0.8 mg/kg.

There is some contradictory evidence in the literature about the effect of GA against the oxidative deterioration of emulsions. Bou et al. (2011) [30] did not see any statistically remarkable effect of GA following its addition in Tween-based sunflower o/w emulsions. Alavi Rafiee et al. (2018) [31] reported that GA exerted a high antioxidant action in the bulk oils but showed lower activity in o/w emulsions, highlighting the critical role of the carboxyl group and the effect of the degree of lipid unsaturation in GA antioxidant activity. Di Mattia et al. (2009) [32] reported that GA contributed to the colloidal stabilisation of the o/w emulsion systems, whilst exhibited a low activity towards secondary oxidation. Zhu et al. (2019) [33], however, observed clear antioxidant effects—in terms of peroxide values and hexanal content- of GA and its alkyl esters in o/w emulsions in the following order of activity: propyl gallate > lauryl gallate > octyl gallate > gallic acid > stearyl gallate. In a study by Wang et al. (2019) [34] GA or its alkyl esters were added in combination with α-tocopherol in o/w emulsions. The results showed that all the tested gallate esters (propyl, octyl and dodecyl gallate) exerted antioxidant activities combined with α-tocopherol, and propyl gallate, with the shortest alkyl chain length, possessed the highest synergistic action. Other researchers have also observed an enhancement of antioxidant activity of esterified GA derivatives in double emulsions by use of encapsulation [35].

### 2.3. Rosmarinic Acid (RA)

RA is an ester of caffeic acid, present as the main phenolic component in several members of the Lamiaceae family including among others: *Rosmarinus officinalis, Origanum* spp., *Perilla* spp., and *Salvia officinialis* [36,37]. A few researchers reported RA as the main phenolic acid of various culinary herbs (oregano, thyme sage, and rosemary) in concentrations varying between 0.05 and 26 g/kg dry weight [38,39]. Additionally, the results of Tsimogiannis et al. [40] indicate an amount of 19.5 g/kg in the leaves of pink savory (*Satureja thymbra* L.).

A body of research has reported antioxidant activities of RA (in terms of both hydroperoxides and volatiles formation) in o/w emulsions based on (i) corn oil and stabilised by various emulsifiers [41]; (ii) Tween-based emulsions prepared with linoleic acid [14] or soybean oil [42]. Bakota et al. (2015) [43] incorporated both pure RA and RA-rich extract (from *Salvia officinalis* leaves), at a concentration of ~30 mg/g, into o/w emulsions and observed that both treatments were effective in suppressing lipid oxidation.

### 2.4. Carnosic Acid (CarA)

CarA is a labdane-type diterpene present in plant species of the Lamiaceae family, such as rosemary and common *salvia* species [44]. CarA is commonly found in the dried leaves of sage in 1.5 to 2.5% concentration [45]. CarA is used as a preservative in food and non-food products, e.g., toothpaste, mouthwash, and chewing gum, since it is endowed with antioxidative and antimicrobial properties [46].

Through the pioneer work of Frankel et al. (1996) [47], CarA was reported to exhibit an antioxidant activity in emulsions that was enhanced at pH 4–5. The following years, several studies used extracts from rosemary and other Lamiaceae family herbs in emulsions and attributed the antioxidant activity mainly to CarA and carnosol [48,49]. Very recently, Lei et al. (2019) [50] have developed a α-tocopherol-based microemulsion aiming to improving the antioxidant stability of CarA and thereby set up a model system for potential applications in bioactive components.

Overall, although this lipid-soluble compound is recognised for its high antioxidative capacities, (which led to many industrial applications in foods and beverages), its mode of action against the oxidation of emulsions have not been yet fully elucidated [51].

### 2.5. Ferulic Acid (FA)

FA is a phenolic acid commonly found in the seeds of coffee, apple, artichoke, peanut, and orange [52]. Flaxseed has been reported as the richest natural source of FA glucoside (4.1 ± 0.2 g/kg) [53]. According to various researchers [54,55], black beans contain FA at an average concentration of 0.8 g/kg. In addition, FA can be found in *Brassica* vegetables and tomatoes [20].

There is a certain body of literature evidence claiming clear antioxidant effects of FA in various o/w systems including: (i) corn oil-based emulsions stabilised with the use of various emulsifiers [56]; (ii) Tween-linoleic acid-based emulsions [14] (iii) in Tween-menhaden o/w emulsions [57]. More recently, Shin et al. (2019) [58] reported that 4-vinylguaiacol (4-VG), (product of FA decarboxylation), exerted a strong antioxidant activity when added at 200 ppm in a 10% o/w emulsion for 50 days. Permin et al. (2019) [59], however, reported that FA showed no antioxidant activity in o/w emulsions rich in ω-3 fatty acids and stabilised by whey proteins.

### 2.6. p-Coumaric Acid (p-CA)

A large number of natural plants sources have been reported to be rich in p-CA such as fungi, peanuts, navy beans, tomatoes, carrots, basil, and garlic [60]. The substance p-CA is abundant in most fruits (especially pears and berries) and cereals [20,61], as well as in honey at a concentration range 1.7–4.7 mg/kg [62]. Kannan et al. (2013) [63], by using HPLC analysis, reported that the extracts of *Halodule pinifolia* and *Clytra rotundata* are rich in p-CA, a fact that may account for their high biological activity. The same authors observed that p-CA is present in high amounts in a few mushroom species. In addition, a few researchers noted that p-CA is present in extracts derived from Amaranth leaves and stem at a concentration range of 28–44 mg/kg [64,65]. Oh et al. (2015) [66] identified p-CA as the main phenolic constituent present in the aqueous extracts of hulled barley (*Hoerdeum vulgare* L.).

In addition, a recent body of research evidence has focused on antioxidant activities of p-CA [67] along with its identification in various natural sources [68]. Very recently, Park et al. (2019) [69] conducted HPLC and NMR analysis of aqueous and ethanolic extracts of roasted rice hulls and identified p-CA, VA, and FA as the dominant phenolic compounds. The authors reported that added roasted rice hull extracts, particular rich in p-CA, protected against the oxidative deterioration of o/w emulsions, at 60 °C.

### 2.7. Vanillic Acid (VA)

VA is a dihydroxybenzoic acid derivative commonly used as a flavoring agent. It is found in several fruits, olives, and cereal grains (e.g., whole wheat), as well as in wine, beer, and cider [70,71]. Kim et al. (2019) [72] performed an identification of the main phenolic constituents in potatoes samples (*Solanum tuberosum* L.) and quantified VA at a concentration between 0.02 and 0.04 g/kg. Espinosa et al. (2015) [73] analysed an extract of red propolis and reported VA as being the major phenolic constituent. VA was also found in fruit extract of the açaí palm plant (*Euterpe oleracea*) [74] and was identified by Zhao et al. [75] in the root of *Angelica sinensis* (an herb indigenous to China) at concentrations between 1.1 and 1.3 g/kg. Furthermore, Radmanesh et al. (2017) [76] reported that VA is present in various botanical sources including *Juglans regia* L., *Chenopodiastrum murale*, orchard grass, and *Melilotus messanensis.*

Keller et al. (2016) [77] observed a strong antioxidant character of VA during autoxidation of Tween 40-based o/w systems, at pH 3.5. Furthermore, Vishnu et al. (2017) [78] evaluated the antioxidant activity of VA grafted chitosan (Va-g-Ch) during the microencapsulation of polyunsaturated fatty acid-rich sardine oil in o/w emulsions. After four weeks of storage, a decrease of peroxide values demonstrated good oxidative stability and encapsulation efficiency of Va-g-Ch.

## 3. Health and Biochemical Properties of Phenolic Acids and Their Natural Extracts

### 3.1. General Health Aspects of Phenolic Acids

Section 2 of the manuscript provided an overview of several natural sources of the phenolic acids while also reported on a number of research findings most of which revealed their clear antioxidant character against the lipid oxidation of o/w emulsions. As also discussed in the introduction of the manuscript, the emulsions could offer useful in vitro model systems as a basis for further investigation of the phenolic activity in interfacial biological systems.

This section focuses on the main target of this review, which is to provide an overview of the latest literature concerning the health properties of the examined phenolic acids. The authors have reviewed a large number of studies investigating into individual phenolic acids and/or their mixtures extracted from natural plant sources. A body of research evidence focuses on the activity of various phenolic acids against cancer and the main mechanisms by which they may exert their effects such as: scavenging of free radicals, induction of enzymes, DNA damage repair, cell proliferation, and apoptosis [82].

Rosa et al. (2016) [83] supported that phenolic acids have been a prime source for the treatment of various forms of cancer, with focus on colon cancer in human colon adenocarcinoma cells. Vinayagam et al. (2016) [84] reviewed the properties of phenolic acids to improve glucose and lipid profiles linked pathologic conditions (diabetes, cardiovascular diseases etc.). A diet rich in phenolic acids has been also reported to protect against certain allergies and slow down the development of Alzheimer’s disease [85].

Table 2 provides an overview of recent in vitro and in vivo clinical studies on the health/biochemical properties of the examined phenolic acids. More specific information per phenolic acid is presented in the following paragraphs.

### 3.2. Caffeic Acid (CA) and its Esters

CA presents a remarkable antioxidant potential and also demonstrates in vitro antimicrobial properties [86]. Further to its well-established antioxidant and anti-aging activities, CA has been reported to own strong antimicrobial properties and protect against dermal diseases [87]. De Oliveira et al. (2012) [88] designed a drug delivery system based on o/w emulsions with CA containing microparticles, developed in order to ensure a prolonged CA release in the target cells and thereby treat the folliculitis skin disease. Similarly, Paulo and Santos (2019) [89] examined how incorporation of caffeic–ethyl cellulose microparticles in skin care products can offer anti-aging protection. Furthermore, a body of recent research evidence has demonstrated that caffeic acid phenyl ester (CAPE) is a natural compound with anticancer activities. The chemical structure of CA (presence of free phenolic hydroxyls) is believed to strongly account for its antioxidant capacities that, in turn, link to certain anti-carcinogenic properties [90]. Dietary supplementation of rats, with CA and CAPE (5 mg/kg body wt subcutaneous or 20 mg/kg oral), was shown to inhibit tumor growth in HCC cells (HepG2) and reduce the tumor invasion at a liver metastatic site [91]. Another clinical study [92] has reported a clear chemoprotective effect of CAPE and its analogs (20 mg/kg body wt) against lipid peroxidation and subsequent cell proliferation of hepatic tumors (HCC) in rats.

Guan et al. (2019) [93] used sucrose fatty acid ester to nano-encapsulate CAPE in aqueous propylene glycol with a temperature-cycle method and reported that nano-encapsulation enhanced cytotoxicity of CAPE against colon cancer HCT-116, and breast cancer MCF-7 cells. Another recent medical study [94] reported clear inhibitory effects of CAPE derivatives against acetylcholinesterase, an enzyme linked with the development of Alzheimer’s disease. CA and its derivatives, such as CAPE, have been reported to act against colon cancer through their cytotoxic to tumors but not to normal cells [95]. In addition, Zhang et al. 2017 [96] examined the action of CA (100 mg/kg) on structural changes caused by HCC in the rat microbiota. The authors concluded that this phenolic compound reduces certain biomarkers that indicate liver injury (among other alanine, transaminase, aspartate aminotransferase, alkaline phosphatase, total bile acid and total cholesterol).

### 3.3. Carnosic Acid (CarA)

Since its first extraction from various natural sources (e.g., *Salvia* and *Rosmarinus* species) and given its well reported functional and antioxidant properties, CarA has been used in a range of cosmetic and pharmaceutical applications [50,51].

Several researchers have focused on the liver protective effect of CarA. In an interesting placebo clinical trial [97] a male *ob/ob* mice (model for NAFLD (non-alcoholic fat liver disease)) followed a diet with CarA for 5 weeks and compared to placebo experienced weight loss and reduced visceral adiposity. The authors concluded that CarA could be considered for the development of new drugs against the NAFLD liver syndrome. Dickmann et al. (2012) [98] explored the hepatotoxicity potential of CarA (at varying concentrations of 4–10 μM) in primary human hepatocytes and microsomes. While CarA did not exhibit any significant time-dependent enzyme inhibition at 4 mM, it even increased enzyme activity at 10 μM, compared with Phenobarbital and Rifampicin drugs. According to the authors, the results indicate potential CarA interaction with drugs, thereby a need for its appropriate safety assessment before its further use as a weight loss supplement.

Bahri et al., 2016 [99] noted that CarA can have a protective effect against chronic neurodegenerative conditions, like Parkinson’s disease, via a mechanism that links to the transcriptional activation of antioxidant Nrf2/ARE pathway.

Einbond et al. (2012) [100], after in vitro experiments in human breast cancer cells, have observed that treatment with CarA at 20 μg/mL resulted in the prevention of ER-negative breast cancer via an activation of expression of antioxidant and apoptosis genes. A more recent study by Solomonov et al. (2018) [101] demonstrated a significant anti-inflammatory effect of CarA combined with astaxanthin and a lycopene-rich tomato extract in a nutrient supplementation.

However, Raes et al. (2015) [45] did not report any effect of CarA, against lipid and protein oxidation in an in vitro simulated gastric digestion model.

### 3.4. Gallic Acid

Over the last few years, a body of research evidence had reported cardioprotective, neuroprotective, and anticancer properties of GA and gallates that are mostly attributed to their antioxidative properties against the reactive oxygen species (ROS) signaling networks [102]. Sourani et al. (2016) [103] reported that GA inhibits proliferation and induces apoptosis in lymphoblastic leukemia cell line. In a very recent study [104] the ability of GA to potentiate the anti-cancer effects of chemotherapeutic drugs (e.g., Paclitaxel, Carboplatin) was examined in human HeLa cells. The authors reported that a Paclitaxel/GA combination could represent a promising alternative with lower side effects for Paclitaxel/Carboplatin combinations in treatment of cervical cancer. Recent pharmacokinetic human and animal clinical studies were based on Chinese GA-based patented medicines but further investigation is needed on the GA kinetic profile after dietary supplementation before drawing any conclusion for its efficacy against pathological conditions [105]. Paolini et al. (2015) [106] performed a study to explore the potential of GA as a promising new anticancer drug. The authors treated T98G human glioblastoma cell lines for 24 h with increasing concentrations of GA (ranging from 1 to 100 μg/mL). According to the results, GA exerts a protective or an anti-proliferative effect on glioma T98G cells via dose-dependent epigenetic regulation mediated by miRNAs.

Yu et al. (2018) [107] contacted a clinical study on myocardial infarcted rats with an oral administration of GA monohydrate at a dose of 50 and 100 mg/kg body wt. The authors observed that myocardial infarction could modify the pharmacokinetic process of GA and thereby determine its potential activity. Similarly, Nwokocha et al. [108] concluded that GA can present negative chronotropic and inotropic effects in isoproterenol induced myocardial damage.

### 3.5. Ferulic Acid (FA)

Over the last few years, a number of clinical studies have demonstrated that FA can exert in vivo antioxidant effects by scavenging free radicals and enhancing the cell stress response through the upregulation of cytoprotective systems [80,109]. Based on its antioxidant and anti-inflammation functions, FA is widely considered as a phenolic compound with well documented protective actions against many pathologic conditions (e.g., types of cancer, cardiovascular diseases, diabetes mellitus and skin problems) [110]. Sgarbossa et al. (2015) [79] reviewed the health benefits of FA and noted its protective role against neurotoxicity based on a number of in vitro and in vivo animal clinical studies. Sung et al. (2014) [111] treated Dawley rats (male, 210–230 g) with FA (100 mg/kg body wt) and reported a clear neuroprotective role. The above-indicated findings recommend the use of FA for drugs development against neurodegenerative diseases, although a few questions are still open before its clinical development and application in patients.

Chowdhury et al. (2016) [112] performed a clinical study that involved oral administration of diabetic rats with FA (at a dose of 50 mg/kg body wt, orally for eight weeks). The authors concluded a protective role of FA against streptozotocin-induced cellular stress in the cardiac tissues. Baeza et al. (2017) [113] reported a strong inhibitory effect of dihydroferulic acid against in vitro platelet activation.

Ambothi et al. (2014) [114] concluded that FA (in the concentration range 10–40 μg/mL) can prevent the ultraviolet-B radiation (290–320 nm) induced oxidative DNA damage in human dermal fibroblasts. The same researchers conducted another clinical study [115] reporting that FA protected against carcinogenesis and tumor formation induced via chronic UVB exposure (180 mJ/cm^2^ for 30 weeks) in the skin of Swiss albino mice. Russo et al. (2017) [116] conducted a population-based case-control study in South Italy to examine any association between dietary phenolic acid consumption and prostate cancer. From a sample of 2044 individuals, 118 histopathological-verified prostate cancer cases were collected, and multivariate logistic regression showed that both CA and FA were associated with reduced risk of this cancer type.

### 3.6. p-Coumaric Acid (p-CA)

p-CA has been reported to decrease the peroxidation of low-density lipoproteins (LDL) and exert anti-mutagenesis, anti-genotoxicity, and anti-microbial activities [117]. Very recently, Ferreira et al. (2019) [118] gave a literature overview of certain biochemical properties of CA (including radical scavenging and tumor suppression activities) that link to its claimed pharmacological effects. Boo (2019) [119] has highlighted the anti-melanogenic effects of p-CA by focusing on its inhibitory action against melanin synthesis as observed in human epidermal melanocytes. Neog and Rasool (2018) [120] supported that dietary p-CA could intervene in the osteoclast formation and thereby alleviate the effect of rheumatoid arthritis, a finding also supported by Trisha (2016) [60].

Pei et al. (2015) [61] focused on p-CA and its conjugates reviewing their dietary sources, and biological activities. The authors concluded that future studies should focus on pharmacokinetic properties of p-CA in order to further promote its use in the food and cosmetic applications. Janicke et al. (2011) [121] treated Caco-2 cells with 150 μM p-CA for 24 h and noticed a protective effect against the development of colon cancer by retarding the cell cycle progression. Ιn addition, Sharma et al. (2017) [122] conducted a study to evaluate the chemo-preventive potential of p-CA in rats challenged with the colon-specific procarcinogen DMH. According to the results, p-CA presented a concentration-dependent anti-carcinogenic effect since it acted more efficiently at a dose of 100 mg/kg body wt, compared to 50 mg/kg body wt. Amalan et al. (2016) [123] reported that p-CA inhibited the development of oxidative stress by increasing the endogenous antioxidant capacity (level of glutathione-GSH) in the livers of diabetic rats. In addition, Vauzour et al. (2010) [124] compared the neuroprotection capacities of various phenolic compounds in primary cultures of mice cortical neuron. The authors concluded a stronger protective effect of p-CA at 1 mM concentration than those of CA and GA. Very recently, Sunitha et al. (2018) [125] reported that p-CA mediated the protection of H9c2 cells from Doxorubicin-induced cardiotoxicity.

### 3.7. Rosmarinic Acid (RA)

Yang et al. (2013) [126] reported the health protective effects of RA on high mobility group box1 (HMGB1) protein-induced inflammation that mediates responses to infection and injury cases. Similarly, Tsung et al. (2013) [127] observed that RA can suppresses *Propionibacterium acnes*–induced inflammatory responses. Concerning the anti-inflammatory mechanism, Ku et al. (2013) [128] observed that RA down-regulates endothelial protein C receptor shedding, in vitro and in vivo. Braidy et al. (2016) [129] investigated whether RA (0.01–0.1 mg/mL) can protect against CTX-mediated toxicity in primary human neurons. According to the results pre-treatment with RA at 0.01 mg/mL (but not higher) exerted a neuroprotective effect, generating significant decrease in CTX-mediated extracellular LDH activity, NAD decline, and DNA damage, compared to CTX treated cells alone.

Nunes et al. (2017) [130] noted that RA displays several health beneficial effects (including antimicrobial and anti-carcionogenic properties) the magnitude of which depends greatly on both its intake and bioavailability. Hossan et al. (2014) [131] have more specifically focused on the anticarcinogenic properties of RA proposing various mechanisms of anticancer activity including antioxidant actions along with proliferation and apoptosis of cancer cells.

Stansbury (2014) [37] summarised the clinical trials that have demonstrated RA activities against allergic immunoglobulin and inflammatory responses of polymorphonuclear leukocytes, thereby being effective in the treatment of allergic disorders. Alagawany et al. (2019) [132] have also reviewed the mode of action, and health benefits of RA.

Domitrović et al. (2013) [133] reported that RA can protect against acute liver damage in intoxicated mice by exerting certain antioxidant, anti-inflammatory, and anti-apoptotic activities. More recently, De Oliveira et al. (2019) [134] examined the protective effects of RA against ethanol-induced DNA damage in mice and reported a clear antigenotoxic capacity in a concentration of 100 mg/kg body wt by using the comet assay. Luno et al. (2014) [135] concluded that RA at 105 μM concentration improves function and in vitro fertilising ability of boar sperm, by inhibiting oxidative stress during cryopreservation. Furthermore, Venkatachalam et al. (2016) [136] investigated the mode and molecular mechanisms that govern the chemoprotective action of RA against colon cancer in rats. The authors reported that supplementation with RA (5–20 mg/kg body wt) protected treated rats from the deleterious effects caused by the colon carcinogenic 1,2-dimethylhydrazine.

### 3.8. Vanillic Acid (VA)

VA has been reported to confer certain health beneficial effects, via antioxidative, anti-mutagenic, anti-cancer, anti-inflammatory, and neuroprotective activities [137,138]. In a recent study [76], male rats (separated in groups of 10) were supplemented with varying concentrations of VA (0–10 mg/kg body wt) for a period of 10 days. The results have shown a clear effect of VA against the risk of myocardial dysfunction. Similarly, Dianat et al. (2014) [139] demonstrated the effectiveness of VA against lipid peroxidation, indicated by malondialdehyde (MDA) reduction, and endogenous antioxidant enzymes improvement, in isolated rat hearts exposed to ischemia-reperfusion. Kim et al. (2010) [140] following a clinical trial in rats reported beneficial effects of VA in the treatment of ulcerative colitis. Erdem et al. (2012) [141] examined the potential effect of VA against mitomycin C-induced genomic damage in human lymphocytes in vitro. Interestingly, VA (at 1 µg/mL) significantly reduced DNA damage cells but at a higher concentration (2 µg/mL) exerted a genotoxic effect on DNA. On the contrary, Krga et al. [142] (2018) reported that VA at 2 μM did not significantly decrease biomarkers of platelet activation development of cardiovascular diseases.

In a clinical trial by Chellammal et al. (2015) [143], five groups of mice were treated as control or active groups supplemented with VA in the concentration range 5–100 mg/kg for 28 days. The results showed that VA at 50 and 100 mg/kg dose significantly (*p* < 0.001) improved the habituation memory, decreased the AChE, corticosterone, and increased the antioxidant capacity of the mice. Furthemore, Yemis et al. (2011) [144] reported a pH-dependent antimicrobial effect of VA that was found to inhibit the growth and heat resistance of Cronobacter bacterial species, a conclusion that could lead to the use of VA for new food storage applications.

### 3.9. Natural Extracts as Mixtures of Phenolic Acids

Nature has generously offered a wide range of herbs (e.g., thyme, oregano, rosemary, sage, mint) that are rich in many phenolic compounds with strong antioxidant biochemical and anti-inflammatory properties [145,146] including protection of DNA from oxidative damage [147]. More specifically, bael (Aegle marmelos) flower (rich in p-CA, CA, and VA) and tulsi (Ocimum tenuiflorum) seeds (rich in GA and p-CA) have been reported to present a strong antioxidant character again DNA damage [148,149].

Findings from recent nutritional intervention studies with natural extracts rich in phenolic acids suggest that they can exert a clear cardio-protective effect through modulations of platelet function [150]. Padmanabhan and Geetha (2015) [151] reported a clear hypo-lipidemic and anti-obesity effect of hydro-alcoholic fruit extract of avocado (particularly rich in GA and VA) in rats fed with high fat diet (co-administered with 100 mg/kg body wt of HFEA for 14 weeks).

Extensive research has been conducted in the last decade about rosemary extracts that are particularly rich in RA and CarA. Chkhikvishvili et al. (2013) [152] demonstrated that a rosemary extract (RE) can protect Jurkat cells from oxidative stress induced by hydrogen peroxide. Very recently, Pérez-Sánchez et al. (2019) [153] investigated the antitumor activity of RE obtained by using supercritical fluid extraction, through its capacity to inhibit various signatures of cancer progression and metastasis. Ulbricht et al. (2010) [154] has published an evidence-based systematic review on RE by examining various aspects of their health properties including also information on their adverse effects and toxicology. In a recent study, Sánchez Salcedo et al. (2015) [155] demonstrated that RE can exert an in vivo anti-tumor action through a reactive oxygen species-initiated cell death. Andrade et al. (2018) [156] reported a clear protective role of RE in preventing colds, rheumatism, and pain of muscles and joints.

De Oliveira et al. (2019) [134] reviewed the in vivo and in vitro studies of *R. officinalis* highlighting the therapeutic and prophylactic effects of RE on some physiological disorders caused by various biochemical agents. Moore et al. (2016) [157] reviewed the phytochemical biological activities and anti-carginogenic properties of *R. officinalis*.

Moreover, p-CA rich methanolic extracts of *Amaranthus spinosus* and of *Amaranthus caudatus L.* were shown to possess significant central and peripheral anti-nociceptive potential and anti-inflammatory activity, in mouse model [36]. Jeong et al. (2017) [158] observed clear therapeutic effects of polyphenolic mixtures (containing among others GA, p-CA and ellagic acid) against cell lung cancer. Hydroxycinnamic acid derivatives of mulberry fruits were reported to increase the production of reactive oxygen species production by acting as pro-oxidants and hence killing the cancer cells [159]. Hilbig et al. (2017) [160] reported that an aqueous extract from pecan nut (particularly rich in GA, CA and VA) showed clear inhibitory effects against breast cancer cell line MCF-7, as well as against tumor growth in Balb-C mice. Simin et al. (2019) [161] provided an overview of the beneficial biological activities of less known wild onions (*A. sect. Codonoprasum*), which are particularly rich in the common phenolic acids. The same group [162] has concluded that a methanolic extract of small yellow onion (*Allium flavum*), particularly rich in FA, *p*-CA, CA, and VA, can exert selective inhibitory action towards cervix epithelioid carcinoma and colon adenocarcinoma cells.

## 4. Conclusions and Future Challenges

Based on the analysis of this manuscript on in vitro and in vivo biochemical activities of phenolic acids, the authors have drawn the following conclusions along with a few recommendations for future investigation in this field:

### 4.1. Activities of Phenolic Acids in O/W Emulsions

Over the last few years, an increasing number of researchers have reported well documented antioxidant activities of naturally occurring phenolic acids in o/w model systems. The findings of the recent studies on phenolic acids antioxidant activity in emulsion formulations could offer a basis for innovative insights in a wide range of food and cosmetic relevant products.

Controlling the interfacial concentrations of antioxidants in o/w emulsions could be regarded as a reasonable approach to monitor more systematically their activity against lipid oxidation Further work in this field could focus on estimating distribution constants of the phenolic acids in emulsions as a factor that would further elucidate and monitor their antioxidant efficiencies in interfacial systems.

Future challenges may include the development of nano-based emulsion systems to enable the delivery of functional bio-constituents (e.g., phenolic acids) and thereby promote their applications in innovative dietary supplements or even drug formulations.

### 4.2. Health Biochemical Properties of Phenolic Acids

This analysis presented a summary of the most recent clinical (mainly animal) studies on phenolic acids. The findings overall offer sufficient evidence to support that each of the examined phenolic acids, through their dietary supplementation, could exert health protective effects against a wide range of pathogenic conditions including cancer, bacterial infections, cardiovascular, inflammatory, and neurodegenerative diseases.

Mixtures of phenolic acids (most commonly present in a wide range of botanical extracts) have been reported by latest research evidence to possess a number of beneficial health properties. The strong antioxidant and biochemical potential of these natural plant preparations may more specifically link to the synergistic effect of their individual phenolic compounds.

Although a few phenolic acids are well-known as efficient bioactive dietary ingredients, their pharmacokinetics and metabolic properties are not fully elucidated yet. This is a factor that limits their current use and therapeutic potential and requires further clinical investigations to support and optimise their future use in nutritional and pharmaceutical applications.

## Figures and Tables

**Table 1:** Natural sources of the examined phenolic acids along with the most recent literature references.

Phenolic Acid Structure	Natural Source	Amount(g/kg, Dry Basis)	Literature
*Caffeic acid* 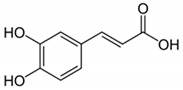	Coffee	0.90 ^a^	[22]
	Blueberry	1.47 ^a^	[22]
	Yerba mate	1.50	[18]
	Banana	0.23–0.31	[70]
	Mango	1.00–1.76	[70]
	*Eucalyptus globulus*	0.2–2.9	[21,79]
*Gallic acid* 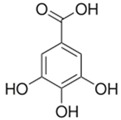	Black tea	0.8	[29]
	banana	1.10–1.24	[70]
	mango	11.45–34.49	[70]
	berries	0.03–0.09 ^a^	[20]
	chestnut	3.50–9.10 ^a^	[20]
*Rosmarinic acid* 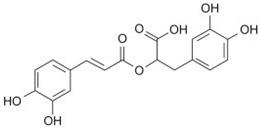	*Rosmarinus officinalis*	0.16–12.86	[38,39]
	*Salvia officinialis*	1.18 ^a^–21.86	[39]
	oregano species	0.05–25.63	[38,39]
	thyme	0.08–6.81	[38,39]
	Sweet basil	10.86	[39]
	Pink savory (*S. thymbra*)	19.50	[40]
*Carnosic acid* 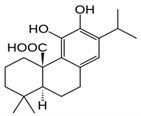	Rosemary leaves	40–100	[51]
	(*R. officinalis*)		
	salvia species	0.1–21.8	[51]
	Sage (*S. officinalis*) leaves	15–25	[45,50]
*Ferulic acid* 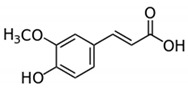	Cereal grains	Up to 2	[80]
	Cell walls of grains	13.51–33.00	[52,81]
	Flaxseed	4.10 (as glucoside)	[53]
	artichoke	2.75	[52,79]
	coffee	0.09–0.14	[53]
	eggplant, redbeet, spinach, peanut	0.07–0.35	[53]
	grapefruit, and orange		
	Banana	0.49–0.53	[70]
	Mango	0.75	[70]
	Beans	0.8	[55]
	Acai (*Euterpe oleracea*) oil	0.10	[71]
*p-Coumaric acid* 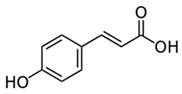	Strawberries	1.11	[20]
	Berries	0.01–0.95	[20,61]
	Pear	0.01–0.45	[61]
	Banana	1.05	[70]
	Mango	0.90	[70]
	Peanuts	1.03 ^a^	[20]
	Onion peel	0.58	[61]
	Honey	0.002–0.005	[62]
	Mushrooms	Traces–3.70	[63]
	*Amaranthus cruentus*	0.028–0.042	[65]
*Vanillic acid* 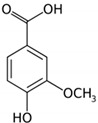	Banana	0.12–0.37	[70]
	Mango	0.47–3.76	[70]
	Acai (*Euterpe oleracea*)	0.002	[74]
	*Angelica sinensis*	1.1–1.3	[75]
	Potato tuber (*Solanum tuberosum*)	0.02-0.04	[72]

^a^: on fresh weight basis.

**Table 2:** Overview of recent in vitro and in vivo clinical studies on the beautiful health/biochemical properties of the examined phenolic acids.

Phenolic Acid.	Experimental Conditions	Conclusion of Study/Health Effect	Reference
*Caffeic acid (CA) & caffeic acid phenyl ester (CAPE)*	Treatment of rats with CA (20 mg/kg body wt).	CA caused suppression of tumor growth in HCC cells (HepG2)/reduction of tumor invasion at liver metastasis.	[91]
	CAPE and its analogs (20 mg/kg body wt) in rats.	CA chemoprotective effect on cell proliferation, p56 activation of hepatic tumors (HCC).	[92]
	CA (100 mg/kg) in the rat microbiota	CA reduce certain biomarkers that indicate liver injury	[96]
*Carnosic acid (CarA*)	Treatment of human breast cancer cells with 20 μg/mL CarA	CarA activated the expression of antioxidant/apoptosis genes resulting in protection against breast cancer.	[100]
	P450 enzyme inhibition was examined in human hepatocytes and microsomes at presence of 4-10 μM of CarA	Increased enzyme activity at 10 mM of CarA, compared to drugs/need for CarA safety assessment before its use against hepatotoxicity	[98]
*Gallic acid (GA)*	Treatment of T98G human cells for 24 h with GA (in the range 1-100 μg/mL).	GA exerts a protective anti-proliferative effect on glioma T98G cells via dose-dependent epigenetic regulation mediated by miRNAs.	[106]
	Oral administration of GA monohydrate (50 and 100 mg/kg body wt) in normal myocardial infracted rats.	Cardioprotective effect of GA.	[107]
*Ferulic acid (FA)*	Treatment of Dawley rats with FA (100 mg/kg body wt).	FA exerted a neuroprotective role by attenuating decreases of peroxiredoxin-2 and thioredoxin levels in neuronal cell injury.	[111]
	Treatment in the skin of salbino mice exposed to UVB (180 mJ/cm^2^) for 30 weeks.	FA protected against carcinogenesis and tumor formation.	[115]
*p-coumaric (p-CA)*	Treatment of Caco-2 cells with 150 μM p-CA for 24 h,	p-CA protective effect against the development of colon cancer retarding the cell cycle progression	[121]
	Treatment of rats (50-200 mg/kg body wt) challenged with colon specific procarcinogen DMH.	p-CA exhibits a significant chemo-preventive potential at 100 mg/kg	[123]
	Treatment of cultures of mice cortical neuron with p-CA (1 mM) against cysteinyldopamine-induced neurotoxicity.	p-CA provided the best neuroprotection, compared to other phenolics (CA and GA).	[124]
*Rosmarinic Acid (RA)*	Study of RA effect (0.01 mg/mL) on cell viability and normal cellular function in human neuronal cells	RA at 0.01 mg/mL (but not higher) exerted a neuroprotective effect generating significant decrease in CTX-mediated extracellular LDH activity compared to control.	[129]
	Treatment of mice (100 mg/kg body wt) and study of the effect on ethanol-induced DNA damage.	Anti-genotoxic capacity of RA against DNA damage (via comet assay)	[134]
	Supplementation of rats with RA (5–20 mg/kg body wt).	RA protected treated rats from the deleterious effects caused by colon carcinogen, 1,2-dimethylhydrazine.	[136]
*Vanillic acid (VA)*	Supplementation of male rats (0–10 mg/kg body wt) for 10 days.	VA was effective against the risk of myocardial dysfunction.	[76]
	In vitro examination of the effect on mitomycin C-induced genomic damage in human lymphocytes.	VA (at 1 µg/mL) significantly reduced DNA damage cells but at a higher (2 µg/mL) itself exerted genotoxic effects on DNA.	[137]
	Supplementation of 5 groups of mice with VA (5–100 mg/kg body wt) for 28 days	VA at 50 and 100 mg/kg dose significantly (*p* < 0.001) improved the habituation memory of mice, and increased the antioxidant capacity.	[143]
